# Chromoplexy: A Pathway to Genomic Complexity and Cancer Development

**DOI:** 10.3390/ijms26083826

**Published:** 2025-04-18

**Authors:** Franck Pellestor, Benjamin Ganne, Jean Baptiste Gaillard, Vincent Gatinois

**Affiliations:** Chromosomal Genetics Unit and Chromostem Research Platform, Department of Molecular Genetics and Cytogenomics, Unique Site of Biology (SUB), University Hospital of Montpellier, 371 Avenue du Doyen Gaston Giraud, 34295 Montpellier Cedex 5, France; benjamin.ganne@chu-montpellier.fr (B.G.); jb-gaillard@chu-montpellier.fr (J.B.G.); v-gatinois@chu-montpellier.fr (V.G.)

**Keywords:** chromoplexy, chromoanagenesis, tumor, prostate cancer, translocation, deletion, gene fusion, punctuated evolution

## Abstract

Chromoplexy is a phenomenon of complex genome rearrangement, occurring during a single cell event and characterized by the formation of chain rearrangements affecting multiple chromosomes. Unlike other genomic rearrangements such as chromothripsis, which involves a single chromosome, chromoplexy affects several chromosomes at once, creating patterns of complex, balanced translocations, and leading to the formation of fusion genes and the simultaneous disruption of several genes. Chromoplexy was first identified in prostate cancers, but it is now observed in various cancers where gene fusions take place. The precise mechanisms behind chromoplexy remain under investigation. The occurrence of these rearrangements follows multiple double-stranded breaks that appear to occur in certain regions or during particular genome configurations (open chromatin, active transcription area), and which lead to an intricate series of inter- and intra-chromosomal translocations and deletions without significant alterations in the number of copies. Although chromoplexy is considered a very early event in oncogenesis, the phenomenon can be repeated and can constitute a mechanism of clonal tumor progression. The occurrence of chromoplexy supports the equilibrium model punctuated by tumor evolution, characterized by periods of relative stability punctuated by sudden and rapid periods of radical genomic changes.

## 1. Introduction

In parallel with the work being carried out on the characterization and etiology of chromothripsis [1,2] and chromoanasynthesis [3,4], a new type of complex «all-in-one» genome reshuffling was described in 2013 in prostate cancer [5] and subsequently identified in other fusion-driven tumors, such as bone and soft tissue tumors [6,7,8]. This new type of massive genomic rearrangement, called chromoplexy, has come to enlarge the rank of complex genomic alterations grouped under the name of chromoanagenesis.

## 2. Definition and Prevalence

Prostate cancer is characterized by numerous genomic alterations (single nucleotide mutations, methylation, copy number changes, microRNAs …), but one of the most characteristic is the occurrence of multiple complex chromosomal rearrangements. As early as 2011, whole-genome sequencing of prostate tumors had shown the frequent occurrence of complex chains of balanced intra- and inter-chromosomal translocations, revealing a distinctive pattern of rearrangements in prostate cancers not previously observed in solid tumors [9]. By sequencing the genomes of 57 prostate tumors and modeling the genesis of recurrent genomic alterations occurring during prostate carcinogenesis, Baca et al. [5] identified these chained rearrangements as a distinct class of catastrophic genomic rearrangements that they named chromoplexy (from the greek *chromo* for chromosome and *pleko* for to weave or to braid). This phenomenon refers to large-scale interdependent rearrangements involving several heterologous chromosomes and leading to the generation of derivative translocated chromosomes with little or no copy-number alterations.

Chromoplexy is now recognized as a milestone in prostate cancer, where up to 90% of tumors display chromoplectic events [5,10]. The CouGar algorithm, a method for characterizing complex genomic rearrangements in cancer genomes, identified the occurrence of chromoplexy in almost 63% of all prostate cancers and in 27% of bladder cancer [11]. Overall, the phenomenon has been observed in about 20% of human cancers, including lymphoid malignancies, Ewing sarcoma, thyroid adenocarcinoma, melanoma, lung cancer, bladder cancer, and neck cancer, suggesting that chromoplexy can occur in a large spectrum of cancer [8,12,13]. Recently, chromoplexy has also been identified as the mechanism underlying neurodevelopmental disorders [14].

The number of rearrangements within a chain is highly variable, ranging from 3 to over 40, with six or more chromosomes involved. In prostate cancer, more than 60% of the tumors involved more than one such chain and 88% of the tumors contained chains with five or more rearrangements [5].

## 3. Mechanisms of Chromoplexy in Prostate Cancer

**Figure 1:**
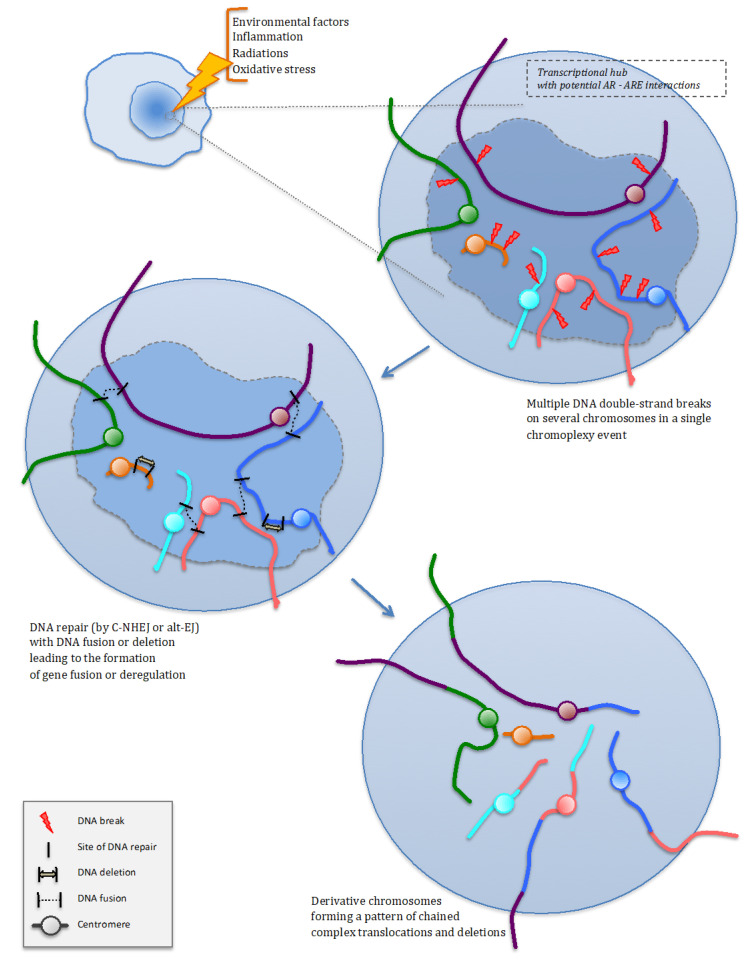
The concept of chromoplexy. In prostate tumors, different factors such as inflammation, radiations, oxidative stress, or environmental factors, can trigger the occurrence of chromoplexy. Several chromosomal domains positioned close together in a nuclear sub-compartment dedicated to transcription (transcriptional hubs), undergo multiple double-strand breaks (DSBs), occurring during a single catastrophic event. These DSBs are repaired by c-NHEJ or alt-EJ mechanisms, which generate interstitial deletions and chromosomal translocations and lead to the formation of fusion genes and deregulation of expression in various genes, thereby promoting tumorigenesis. The occurrence of these multiple chromosomal rearrangements results in the formation of derivative chromosomes forming a pattern of chained intra- and interchromosomal complex genomic rearrangements (mainly translocations and deletions). Six chromosomes are schematically represented, each in a different color.

The mechanistic origins of chromoplexy are not fully understood. Chromoplexy appears to be a highly complex genomic event, the formation of which involves multiple double-strand breaks (DSBs) occurring in different regions of the genome (Figure 1).

Various causal factors have been suggested to explain the initiation of chromoplexy in somatic cells and the gene fusion events that characterize it. DSBs, which are a central trigger for gene fusions, can be caused by ionizing radiation, oxidative stress, dysfunction of topoisomerase II, or inflammation-induced by reactive oxygen species (ROS). The prostate gland, rich in oxidative metabolic processes, may be particularly sensitive to these factors [15,16]. Chronic inflammation (e.g., prostatitis) can also generate ROS, cytokines, and enzymes like activation-induced cytidine deaminase (AID), that promote genomic instability and DNA breaks. The phenomenon of gene fusion following DSBs is also undoubtedly facilitated by the physical proximity of the genes involved, as is the case for the *TMPRSS2* and *ERG* genes on chromosome 21 [17]. In addition, environmental and lifestyle factors such as high-fat diets, endocrine disruptors or exposure to genotoxic chemicals could influence oxidative stress, which is relevant to the mechanisms mentioned above [18].

Whole genome sequencing studies of prostate tumors have revealed a remarkable feature of chromoplexy, i.e., recurrent gene fusions, with the generation of chimeric chromosomes through “close chains” of broken and rejoined chromosome segments. In prostate cancer, these genome fusion events, spread over several chromosomes, typically involve the fusion of various genes with genes from the *ETS* (E-26 Transformation Specific) family of transcription factors, in particular *ERG*, *ETV1*, *ETV4* and *ETV5* genes, and produce “poly-gene” fusion events that can disrupt multiple genes simultaneously and activate oncogenes. The most prevalent *ETS* gene rearrangement is the fusion of the proto-oncogene *ERG* (ETS-Related Gene) with the gene *TMPRSS2* (Transmembrane Protease, Serine 2), both located in 3 Mb apart on human chromosome 21 (on chromosomal bands 21q22.2 for *ERG* and 21q22.3 for *TMPRSS2*). It has been postulated that the short distance between the *TMPRSS2* and *ERG* genes on the chromosome 21 could account for the higher frequency of *TMPRSS2:ERG* fusions in prostate cancer [19]. The high prevalence of this fusion suggests that this chromosomal region could be a hot spot for rearrangement in prostate cancer. *ERG* is a member of the *ETS* family of transcription factors, which are key regulators of embryonic development, differentiation, cell proliferation, inflammation and apoptosis. *ERG* transcription factors can act as transcriptional activators and/or repressors, depending on the target gene, post-translational modifications and interactions with protein cofactors [20]. *TMPRSS2* is an androgen responsive transmembrane serine protease, predominantly expressed in the luminal cell of the prostate epithelium [21]. The expression of the *TMPRSS2* protein at the cell surface regulates cell–cell and cell–matrix interactions.

In prostate cancer, the most common gene fusion event is the fusion between the promoter region of *TMPRSS2* and the coding region of the *ERG* gene. The *TMPRSS2* promoter contains androgen-sensitive elements, and the expression of this gene is hormone-dependent. Because of this characteristic, the expression of the *TMPRSS2:ERG* fusion gene leads to the overexpression of the *ERG* transcription factor in the presence of androgens in about 50% of prostate tumors [22]. Numerous *TMPRSS2:ERG* fusion transcripts have been identified, which can be generated by different combinations of exons from the *TMPRSS2* and *ERG* genes [23,24,25]. Similar fusional rearrangements promote aberrant expressions of the *ETS* transcription factors *ETV1* and *ETV4* in another 10% of cases [26]. Together, the chromoplectic rearrangements of these three *ETS* factors are considered to be early driver events that promote tumorigenesis in the majority of prostate cancers [27].

**Figure 2:**
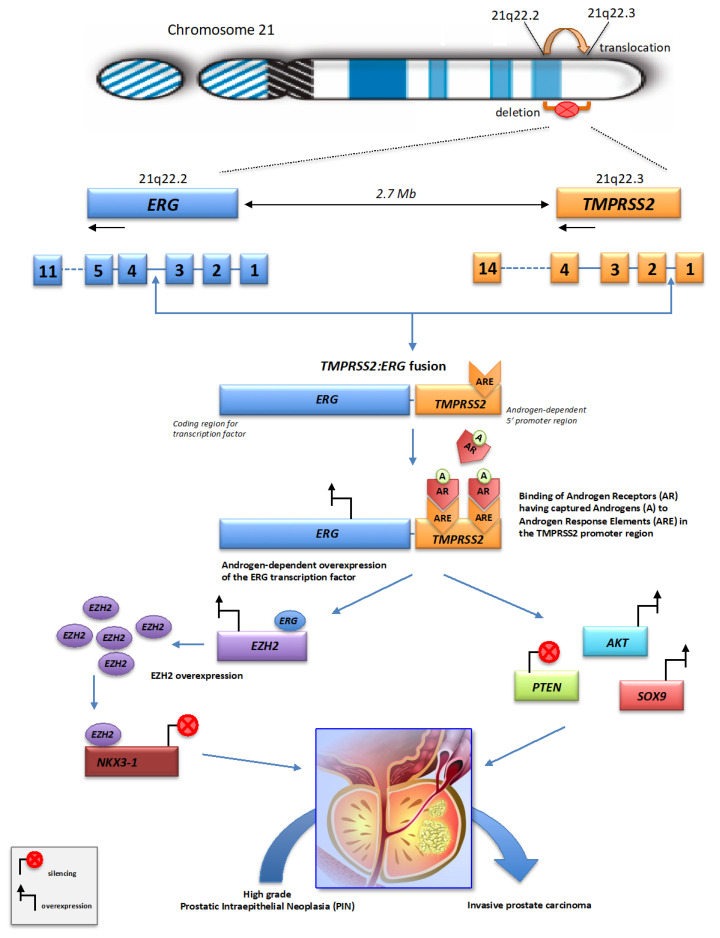
The *TMPRSS2:ERG* fusion in prostate cancer. The fusion of *TMPRSS2* and *ERG* can occur by interstitial deletion or translocation between the 5′ promoter region of *TMPRSS2* and the coding region of the *ERG* gene. Androgen receptors (ARs) having captured androgens, can bind to androgen response elements (AREs) located on the promoter region of *TMPRSS2*, thereby bringing *ERG* under transcriptional regulation of androgen and leading to its overexpression. This upregulation of *ERG* leads to overexpression of the *EZH2* gene and subsequent silencing of the *NKX3-1* gene, as well as the up- or down-regulation of several genes involved in various cellular processes, such as *AKT* and *SOX9*. *ERG* overexpression is also associated with loss of the tumor suppressor *PTEN*. This synergistic action facilitates the progression of prostatic intraepithelial neoplasia (PIN) to invasive carcinoma.

The gene fusions can occur as the result of either interstitial deletions or interstitial translocations [28,29] (Figure 2). These two mechanisms of chromosomal rearrangement may coexist within the same prostate tumor [30]. The fusion breakpoints mostly exhibit precise joins with neither overlapping nor intervening sequence at the rearrangement junctions. This results in derivative chromosomes that maintain a largely balanced DNA content. Alternatively, substantial deletions can be observed at the junctions of the chained rearrangements, creating “deletion bridges” that spanned the sequences between breakpoints from two different fusions. DSBs can be repaired by canonical non-homologous end-joining (c-NHEJ) or alternative end-joining (alt-EJ) repair processes, which can promote the formation of structural chromosomal alterations if multiple DSBs have to be repaired [10,31,32].

In tumors without these specific fusions, closed chains of disruptive rearrangements can regulate other tumor suppressor genes, such as *PTEN*, *TP53*, *AKT*, *MAP2KA*, *CHD1*, and *NKX3-1*, which also promote the progression of prostate cancer [5,9,33]. This indicates that chromoplexy may dysregulate multiple genes in parallel to drive prostate tumorigenesis.

Due to the prevalence of *ETS*-associated chromoplectic rearrangements, two distinct subtypes of prostate tumor are distinguished. Chromoplexy involving oncogene *ETS* fusion (*ETS*+) represents the largest molecular subtype of prostate cancer. This group of tumors displays significantly more inter-chromosomal rearrangements than tumors without *ETS* gene fusion (*ETS*-) and involves a greater number of chromosomes in a single event. In contrast, chromoplexy in *ETS*- tumors displays similar features to chromothripsis, with up to seven-fold more rearrangements than the average [5]. Among the *ETS*- tumors, some display an overexpression of the *EZH2* gene, independent of *ERG* expression. Other tumors harbor disruptive rearrangements or deletions of the *CHD1* gene, a chromatin-remodeling factor whose inactivation can prevent *ERG*-fusion occurrence [34,35]. Tumors with deletion of *CHD1* display an excess of intra-chromosomal chained rearrangements and deletions. This raises the possibility that *CHD1* deletion may contribute to an alternative pattern of genome instability in tumors.

Although chained translocations could theoretically arise by sequential process through multiple cell generations, such a mechanism seems unlikely because it fails to account for the proximity and the interlocking nature of the complex chains of rearrangements observed in prostate tumors. Statistically, it is unlikely that closed chain patterns of rearrangements would occur independently of each other. Their formation must be spatially and temporally coordinated. Such a process implies phenomena of chromosome clustering or chromosome migration within the nucleus before undergoing rearrangements, in line with the movement observed within nuclear chromosome territories [36,37] and the existence of topologically associating domains (TADs) [38,39].

Chromoplectic rearrangement breakpoints are associated with active transcription and open chromatin configuration. In addition, genomic regions implicated in chromoplexy are often found in early replicating regions and are rich in expressed genes [8]. The occurrence of complex chains of chromoplectic rearrangements involving *TMPRSS2:ETS* fusions reflects a process of gene restructuring and DNA injuries in distinct nuclear sub-compartments containing RNA polymerases and thus forming transcriptional hubs where loci from multiple chromosomes come to cluster [40]. In the 5′ promoter domain of *TMPRSS2*, the breakpoints are preferentially localized close to sites called androgen response elements (AREs) on which the androgen receptors (AR) bind as transcription factors [41,42]. ARs are nuclear transcription factors, which mediate the action of androgens (testosterone and dihydrotestosterone) [43]. The fusion process renders *ERG* under the control of androgen responsive (Figure 2). The androgen-induced binding of AR to ARE sites of *TMPRSS2* leads to changes in chromosomal conformation resulting in looped chromatin necessary for transcription coordination [17,44].

These data indicate that AR-ARE complexes could be area prone to genomic rearrangements through transcriptional stress. Indeed, androgen signaling favors the co-recruitment of AR and topoisomerase II b (TOP2b) at *TMPRSS2:ERG* fusion sites, triggering TOP2b-mediated DNA double strand breaks [17] within or near transcriptional hubs. The result is the bringing together of different sections of the genome into proximity, which upon breakage can reconnect via the formation of intra- or inter-chromosomal rearrangements. *TMPRSS2:ERG* fusion is also controlled by androgen-dependent methylation of the *KDM1*A gene by the histone methyltransferase *EHMT2*. This event is necessary for *TMPRSS2* enhancer-breakpoint loop formation [45]. Genomics and proteomics analysis have evidenced the co-localization of *KDM1A* and AR in prostate tumor cells [46].

The *ERG* overexpression induced by *TMPRSS2:ERG* fusion initiates a cascade of events (Figure 2), in particular the overexpression of *EZH2*, a methyltransferase involved in silencing of tumor suppressor gene [41,47], and the decreased expression of *NKX3-1*, an androgen-regulated prostate specific gene which acts as a negative regulator of epithelial cell growth in prostate tissue [48]. The overexpressed *ERG* protein functions as a transcription factor regulating the expression of genes involved in various tumor-related cellular processes, such as proliferation, differentiation, metastasis, and apoptosis. *ERG* and *EZH2* interact physically, and this functional *ERG*-*EZH2* interaction enhances *ERG* transcriptional activity, producing profound changes in the transcriptional activity of prostate epithelial cells and sustaining prostate cancer progression [49]. The *ERG* overexpression is also associated with increased expression of *SOX9*, a transcription factor required for prostate development whose activation may mediate the invasive phenotype caused by *ERG* overexpression. Studies have indicated that *TMPRSS2:ERG* fusion may co-operate with the loss of the tumor suppressor *PTEN* and the concomitant activation of *AKT* to promote the progression of prostate cancer from high-grade prostatic intraepithelial neoplasia (PIN) to invasive prostate carcinoma [50,51]. The *TMPRSS2:ERG* fusion process has also been described as involved in the regulation of long non-coding RNAs (lncRNAs), which are known to play an important role in the regulation of gene expression and to be deregulated in several types of cancer, including prostate cancer [52,53].

Sequential events of chromoplexy can be detected at a clonal or subclonal level in prostate cancer [54], indicating that the prostate tumor may undergo multiple rounds of chromoplexy, triggering the emergence of tumor subclones during the cancer progression. This is consistent with the concept of intra-tumoral heterogeneity, caused by multifocality in prostate cancer and contributing to the aggressive behavior of high-grade prostate tumor [55].

These data provide a mechanistic understanding of chromoplexy-linked genomic reorganization process driving rapid evolution in prostate cancer. They contribute to deciphering the mechanism of carcinogenesis and metastatic progression of prostate cancer.

## 4. Involvement of Chromoplexy in Other Cancers

In Ewing sarcoma, the chromoplexy phenomenon is manifested by the occurrence of chromosomal translocations between the *EWSR1* gene (Ewing Sarcoma breakpoint region 1 protein) located on chromosome 22 (band 22q12.2) and genes belonging to the *ETS* family of transcription factors, in particular *FLI1* (located on chromosome 11, band 11q24.3) in 90% of cases [56], and *ERG* in 5 to 10% of cases [57]. These rearrangements observed in 40 to 60% of tumors [8,58] lead to the formation of chimeric proteins, generally *EWS:FLI1* or *EWS:ERG*, in which the C-terminus of the EWS protein is replaced by the *ETS* DNA-binding domain of an *ETS* family transcription factor [57]. The involvement of *EWSR1* gene accounts for 76 to 93% of chromoplectic events detected in Ewing Sarcomas. In cases involving the *ERG* gene, this leads to a loss of endogenous activity of the *ERG* gene promoter, causing deregulation of the *ERG* transcription factor and its target genes. The particular *EWSR1-ERG* translocation is characterized by the formation of acentric/dicentric chromosomes. The unstable derivative chromosomes could then undergo additional rearrangements, in a more complex chain loop, making it possible to stabilize the *EWSR1:ERG* fusion, thus increasing the occurrence of chromoplexy events. The *EWSR1-ETS* Ewing loops appear to be less complex than *TMPRSS2:ERG* prostate cancer loops with fewer rearrangements (two to ten rearrangements in one or two loops). Deletion bridges are also observed in 60% of chromoplectic Ewing sarcoma, creating further oncogenic disruptions [8]. Given their tight clustering and low copy number alterations, *EWSR1-ETS* loops are likely to occur in a burst of rearrangements. According to all these data, it appears that over 90% of the chromoplectic rearrangements found in Ewing sarcomas are interchromosomal translocation involving the *EWSR1* and *FLI1* genes.

Chromoplexy therefore plays a crucial role in Ewing sarcoma initiation, not only as a secondary event, but as a major event in the early cell transformation process [58]. Chromoplexy marks an aggressive form of Ewing sarcoma. However, the biological consequences of this multigene disruption remain poorly defined. The particularity of chromoplexy lies in the fact that it occurs within the somatic cell, contributing directly to the initiation and progression of tumors. Recently, sarcomagenesis phenomena, including *EWSR1:FLI1* and *EWSRI:ERG* fusions have been modeled, making it possible to study the mechanisms linked to chromoplexy and the way in which cells adapt to such catastrophic burst of complex rearrangements [59].

Complex chromoplexy-linked rearrangements have also been identified in aggressive forms of mantle cell lymphomas, with the presence of chained reciprocal rejoining genomic events and fusions of gene such as *ANK2* and *SOX5*, in a context of chromatin organization that suggest that genomic regions involved were physically proximal and interacting [60]. Similar closed-chain patterns of complex genomic rearrangements, involving the *BRD3:4-NUT* oncogenic fusion and extensive chromatin remodeling, occurring in a single catastrophic event, have been observed in nuclear protein in testis (NUT) midline carcinoma [61]. Similarly, the study of the mutational processes operating in lung adenocarcinomas has revealed the formation of driver fusion oncogenes generated from chromoplexy-related genomic rearrangements, as initiating events of malignant transformation [62]. More recently, it was reported that chromoplexy could occur in late stages of hepatocarcinogenesis [63]. The aggressive transformation of chronic lymphocytic leukemia into Richter syndrome is also marked by alterations in chromatin accessibility and the occurrence of chromoplectic events [64].

## 5. Comparison with Chromothripsis

The precise demarcation between chromoplexis and chromothripsis is still rather vague and it can be tricky to define the specific characteristics of each of the two phenomena (Table 1).

Chromothripsis is a catastrophic phenomenon that has been identified in cancer genomes, in patients with congenital diseases, in embryos and in the genomes of healthy individuals [65,66,67,68,69,70,71]. To date, chromoplexy has mainly been documented in cancer.

Chromothripsis events are generally localized to one or two chromosomes, with the generation of numerous structural rearrangements. In comparison, chromoplexy affects several chromosomes at once, creating patterns of complex, balanced translocations, but the concerned chromosomes show fewer rearrangements than those involved in chromothripsis (tens vs. hundreds).

Chromothripsis is typically a single event, which often gives rise to losses of acentric chromosomal segments leading to multiple alterations between the losses and diploid chromosomal segments in which heterozygosity is conserved. On the other hand, chromoplexy occurs as closed chains with nearly precise junctions and almost no deletions. Consequently, the characteristic copy-number oscillation observed on chromothripsis is not found for chromoplexy. Furthermore, in chromothripsis, chromosome fragments are joined together in random order and orientation, whereas in chromoplexy, the original chromosomal orientation is retained.

In cancer, the observation of chromothripsis events in all tumor cells, not just subclones, supports the hypothesis that chromothripsis is typically the result of a single catastrophic cellular event during cancer progression [72]. In contrast, chromoplexy can constitute a single and chaotic cellular event that generates multiple breakpoints simultaneously, but it can also occur sequentially over several cell cycles or throughout tumor progression at clonal or subclonal level, as observed in prostate cancer [5,54].

Chromoplexy and chromothripsis are not entirely exclusive. Both can occur concurrently or asynchronously in the same cell, generating diverse patterns of chromosomal complexity [73]. Some of the rearrangements observed may present intermediate characteristics between chromoplexy and chromothripsis [55]. Chains of translocations generated by chromoplexy can also produce instable karyotypes that subsequently trigger chromothripsis events [62,74].

## 6. Conclusions

Chromoplexy is a complex chromosomal mechanism underlying carcinogenesis. The detailed analysis of this phenomenon in prostate cancer indicates that chromoplexy plays a crucial role in tumor initiation and subsequent clonal evolution. Chromoplectic events can disrupt tumor suppressor genes or create oncogenic fusions, accelerating cancer progression while also contributing to tumor heterogeneity and therapeutic resistance [75]. By impacting multiple cancer-related genes, it provides a selective or proliferative advantage to cancer cells and promotes the early progression of prostate cancer. It now appears as a major event in the cell transformation process in a wide spectrum of cancers. The high prevalence of recurrent gene fusions that characterize it highlights the fact that chromosomal rearrangements are critical initiating events in the evolution of cancer.

The occurrence of chromoplexy supports the punctuated equilibrium model as a mechanism for rapid cancer evolution. In contrast to the classical view of tumorigenesis based on a gradual accumulation of cancer-promoting mutations, the large-scale genomic rearrangements of chromoplexy are in good agreement with the concept of punctuated evolution of tumor characterized by periods of stability interrupted by rapid and radical bursts of genomic alteration resulting from successive round of chromoplexy or the sequential occurrence of chromoplexy and chromothripsis events [5,76]. In prostate cancer, Baca et al. [5] proposed a model of punctuated tumor evolution in which a tumor genome may sustain considerable damage over several sequential and punctuated events that can be attributed to successive cycles of chromoplexy or the sequential occurrence of chromoplexy and chromothripsis.

## 7. Future Perspectives

Research on the mechanisms of chromoplexy is essential to understand its role in cancer evolution and therapeutic resistance. Thus, accurate knowledge of the initiating events that trigger the *TMPRSS2*:*ERG* fusion will give deeper insight into early prostate tumorigenesis. Looking ahead, future research should benefit from advances in single-cell sequencing, long-read genome technologies (like Oxford Nanopore and PacBio), and AI-driven structural variant analysis [77,78]. These tools can help to better identify the biological triggers of chromoplexy and to unravel the temporal evolution of chromoplectic events. Additionally, integrating chromoplexy mapping with 3D genome architecture studies may help clarify how spatial genome organization contributes to the occurrence of these complex rearrangements. Advances in high-throughput sequencing technologies and single-cell genomics have already made it possible to identify these rearrangements with greater precision and to distinguish the clonal evolution from transient rearrangements, thus opening the door to early cancer diagnostics and more personalized approaches to treatment [12,79]. The liquid biopsy has become a highly sensitive and promising approach to molecular diagnostic that is beginning to be used to detect biomarkers in blood and urine, notably ERG and EST transcription factors in early prostate cancer [80,81,82].

From a therapeutic perspective, the intricate rearrangements caused by chromoplexy present both challenges and opportunities. On the one hand, they complicate the genomic landscape, making targeted therapy more difficult. On the other hand, the unique structural variants created by chromoplexy may serve as biomarkers, paving the way for personalized treatments, including immunotherapy and therapies targeting DNA repair mechanisms [83,84]. The challenge lies in targeting such dynamic and complex rearrangements. However, the rearranged regions may generate neoantigens—abnormal peptides recognizable by the immune system—providing a rationale for personalized immunotherapies. Furthermore, *PARP* inhibitors (PARPi) and DNA damage response (DDR) inhibitors can be effective against tumors with high levels of genomic instability resulting from chromoplexy. Recent studies have shown that *TMPRSS2:ERG* gene transcript product could be used as a PARPi resistance biomarker [85]. The interplay between genomic instability, the occurrence of chromoplexy, and the tumor microenvironment in modulating the outcome of treatment also deserves further exploration if we are to understand the root causes of genomic chaos in cancer [86,87]. Deeper integration of chromoplexy signatures into clinical genomic profiling could ultimately guide therapeutic decisions, helping to predict resistance patterns and adapt combination therapies in cancers such as prostate cancer and Ewing sarcoma [88,89].

Beyond the impact of chromoplexy as a pathogenic process, an interesting question is the potential driving role of the chromoplexy phenomenon in the evolution of species and their genomes. Over the last decade, numerous studies have shown how chromosomal and genomic alterations can have a considerable impact on developmental evolution [76,90]. Understanding the mechanistic basis of chromoplexy should allow us to pinpoint the possible role of this chaotic phenomenon in genome evolution.

## Figures and Tables

**Table 1:** The key differences between chromothripsis and chromoplexy.

Key-Features	Chromothripsis	Chromoplexy
Number of events	Single	single or sequential
Number of chromosomes involved	Usually 1 or 2 (up to 4)	Multiple (3 to 7)
Rearrangements	Balanced rearrangements, deletions, duplications, insertions… ± extra-chromosomal circular elements	Inter- or intra-chromosomal balanced translocations, ± deletions
Breakpoints	Numerous (up to 100) Clustering of breakpoints	On average from 5 to 40
Breakpoint signature	Blunt ends (possibly small insertions)	Blunt ends (possibly small insertions)
Repair mechanisms	NHEJ	NHEJ/alt-EJ
Junction and order of chromosomal fragments	Random order and random orientation	Conservation of the original chromosome orientation
Copy number state	Oscillating pattern between 2 copy number states	No copy number alterations

## Data Availability

No applicable.
